# Performance of the novel ANTWERP score in predicting heart function improvement after atrial fibrillation ablation in Asian patients with heart failure

**DOI:** 10.1002/joa3.13162

**Published:** 2024-10-18

**Authors:** Lo‐Chieh Ling, Ting‐Yung Chang, Yenn‐Jiang Lin, Chin‐Yu Lin, Shih‐Lin Chang, Li‐Wei Lo, Yu‐Feng Hu, Fa‐Po Chung, Shih‐Ann Chen

**Affiliations:** ^1^ Division of Cardiology, Department of Medicine, Heart Rhythm Center Taipei Veterans General Hospital Taipei Taiwan; ^2^ Division of Cardiology Taipei Veterans General Hospital Taipei Taiwan; ^3^ Institute of Clinical Medicine and Cardiovascular Research Institute National Yang‐Ming Chiao Tung University Taipei Taiwan; ^4^ National Taipei University of Nursing and Health Sciences Taipei Taiwan; ^5^ Department of Internal Medicine Taichung Veterans General Hospital Taichung Taiwan; ^6^ National Chung Hsing University Taichung Taiwan

**Keywords:** ablation, ANTWERP score, atrial fibrillation, ejection fraction, heart failure

## Abstract

**Background:**

Previous research has demonstrated that atrial fibrillation (AF) ablation improves heart function variably among patients. We proposed that the ANTWERP score, which was validated in a European group of patients with low left ventricular ejection fraction (LVEF) who had AF ablation, would be valid in an Asian group as well. The purpose of the study is to examine how well a new scoring system (the ANTWERP score) can predict heart function improvement after atrial fibrillation ablation in Asian patients with heart failure.

**Methods:**

A retrospective review was conducted on patients (*n* = 84) undergoing AF ablation between January 2019 and June 2022. Initial diagnoses for impaired LV systolic function were confirmed by echocardiography. Patients meeting the “2021 Universal Definition of HF” criteria for LVEF recovery were classified as “responders.”

**Results:**

Similarities were observed between responders and nonresponders regarding comorbidities, AF type, and LVEF, except for the left ventricular internal diameter in diastole. A higher percentage of responders had an ANTWERP score ≤2 (87.8%) compared to those with a score >2 (55.6%). LVEF improvement was notably higher in the former group (+14.8% vs. +9.4%, *p* = .043). Atrial reverse remodeling and recurrent atrial arrhythmia rates were similar across groups.

**Conclusion:**

The conclusion of the study was that the ANTWERP score effectively predicted LVEF improvement after atrial fibrillation ablation in the Asian population and that this scoring system could be used to guide clinical decisions and prognosis prediction.

## Graphical abstract

Central illustration: The ANTWERP Score in Predicting LVEF Recovery after AF Ablation in the Asian Population.

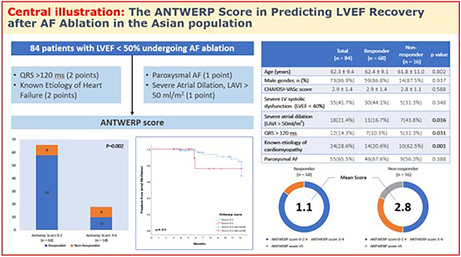

## 1 INTRODUCTION

Atrial fibrillation (AF) and heart failure (HF) frequently coexist, exacerbating each other's progression and resulting in increased symptoms, morbidity, and mortality.^1^, ^2^, ^3^ Subsequent analysis of data from the CASTLE‐AF trial, which compared Catheter Ablation versus Standard Conventional Treatment in patients with left ventricular dysfunction and AF, indicated that maintaining sinus rhythm (SR) and reducing the burden of AF (to <50%) could enhance patient outcomes in the context of HF.^4^ Over the past two decades, catheter ablation has emerged as a viable therapeutic strategy for managing AF.^5^ A meta‐analysis of randomized controlled trials assessed the effectiveness and results of catheter ablation versus medication therapy in HF patients with AF.^6^ The findings revealed that AF ablation, when compared to standard pharmacological treatment, significantly enhances outcomes across multiple measures, including all‐cause mortality, rates of hospitalization for HF, left ventricular systolic function, distances achieved in the 6‐min walk test (6‐MWT), peak oxygen consumption (VO2max), and overall quality of life.^7^, ^8^, ^9^ AF ablation has been endorsed with a Class IIa recommendation by the 2020 European Society of Cardiology guidelines for rhythm control in patients with HF with reduced ejection fraction (HFrEF) and received the same recommendation for patients with symptomatic HF in the 2022 guidelines issued by the American Heart Association/American College of Cardiology/Heart Failure Society of America for heart failure management. Despite these endorsements, the Atrial Fibrillation Management in Congestive Heart Failure with Ablation (AMICA) trial, recently published, failed to confirm the benefits of AF ablation in HF patients with severely reduced left ventricular ejection fraction (LVEF).^10^ Consequently, AF ablation is observed to improve LVEF in a variable proportion of HF patients. A new diagnostic tool, the ANTWERP score, which utilizes a straightforward four‐parameter system for the personalized evaluation of HF patients with reduced LVEF undergoing AF ablation, was recently introduced.^11^ This scoring system was shown to correlate clinical and echocardiographic parameters (AF classification, left atrial volume, HF etiology, and QRS duration) with LVEF recovery post‐AF ablation. This correlation has been substantiated in a large multi‐center study across Europe.^12^ In this investigation, patients presenting ANTWERP scores below 2 points demonstrated a 93% likelihood of recovering LVEF, starkly contrasting to a mere 24% among those with scores exceeding 3 points. Nonetheless, the efficacy of this scoring system has not yet been evaluated in Asian populations. This study is designed to examine the reliability and relevance of these established scores within an Asian cohort, highlighting the imperative to verify the score's precision and consistency across diverse ethnic groups.

## 2 METHODS

### 2.1 Study population

**FIGURE 1:**
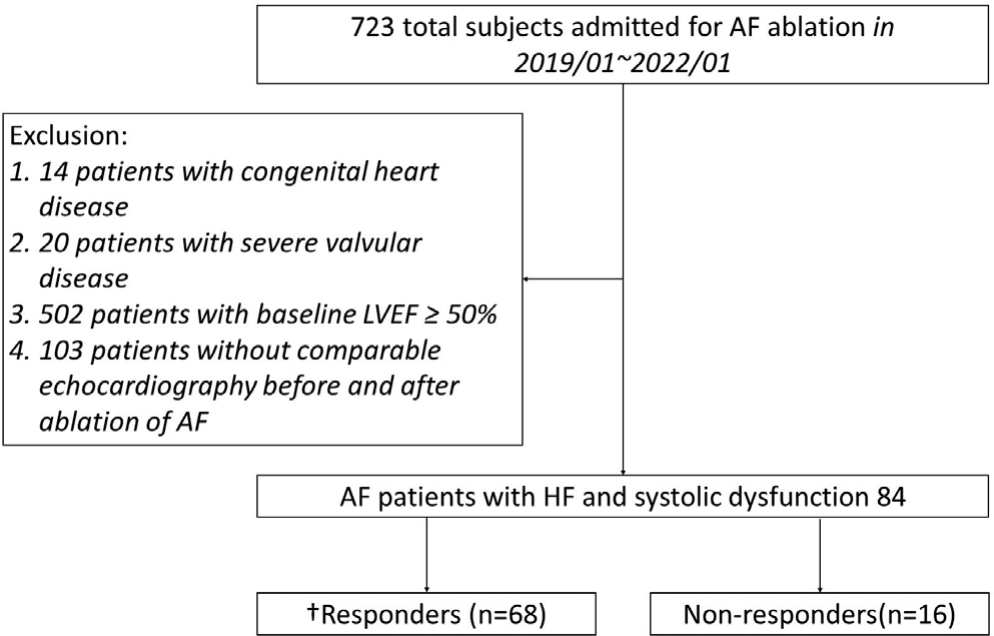
Patient allocation and analysis. AF, Atrial fibrillation; LVEF, Left ventricular ejection fraction; HF, Heart failure. Responders were identified (i) in case of baseline LVEF between 40% and 50% (HF with mildly reduced ejection fraction): LVEF improvement to more than ≥50% and (ii) in case of baseline LVEF <40% (HF with reduced ejection fraction): ≥10% absolute increase from baseline LVEF, and LVEF >40%.

Between January 2019 and June 2022, we retrospectively screened 723 patients admitted to Taipei Veterans General Hospital for AF ablation. The exclusion criteria were^1^ congenital heart disease,^2^ severe valvular disease,^3^ patients with LVEF ≥50%, and^4^ patients lacking comparable echocardiography before and after ablation (Figure 1). AF was defined according to the 2017 Heart Rhythm Society Expert consensus document statement.^13^

### 2.2 Study design

This retrospective study was carried out at Taipei Veterans General Hospital in Taipei following approval by the institutional review board of the same hospital. (IRB: 2021‐11‐015BC, 2022‐06‐001BC). The investigation conforms with the principles outlined in the Declaration of Helsinki. Both baseline data and echocardiography parameters were collected from the medical records of each patient. All patients had optimized HF therapy at the time of AF ablation. The study's primary endpoint is the recovery of LVEF within 1 year following the initial procedure. Patients with recovery with LVEF were labeled as responders according to the “Universal Definition and Classification of HF.”^14^ Responders were identified if (i) in case of baseline LVEF between 40% and 50% (HF with mildly reduced ejection fraction): LVEF improvement to more than ≥50% and (ii) in case of baseline LVEF <40% (HF with reduced ejection fraction): ≥10% absolute increase from baseline LVEF, and LVEF >40%. Others were labeled as nonresponders. Atrial‐arrhythmia recurrence‐free survival and cardiac remodeling were also collected. Patients underwent evaluations between 3 and 12 months after the ablation or upon the onset of symptoms during the first year. An ambulatory 24‐h Holter monitor and echocardiography were conducted during each assessment.

Ischemic cardiomyopathy was characterized by systolic dysfunction resulting from myocardial damage and ischemia.^15^ Valvular heart disease was defined by the presence of severe valvular dysfunction or a history of valve repair or replacement.^16^ For nonischemic cardiomyopathy, if a specific cause, such as a genetic mutation or other identifiable factors, was determined, it was classified as having a “known etiology.” This category included conditions such as Fabry's disease, dilated cardiomyopathy, hypertrophic cardiomyopathy, and myocarditis, following established guidelines.^17^, ^18^ If no clear cause was identified, the patient was categorized under “unknown etiology.”

### 2.3 Transthoracic echocardiogram

The baseline echocardiography was performed within 1 year before ablation, and the follow‐up echocardiography was performed at least 3 months after ablation. Two‐dimensional images were obtained using an EPIQ CVx (Philips Healthcare, Andover, USA) or VividTM E95 (GE Healthcare, Horten, Norway) with a 2.5–5 MHz Doppler transducer.^19^ According to the American Society of Echocardiography recommendations, the left atrium (LA) diameter was measured during M‐mode in the parasternal long‐axis view. Simpson's rule was modified for LVEF calculation.

### 2.4 Electrophysiology study, mapping, and ablation strategy

We employed a stepwise catheter ablation procedure, described in detail in our previous studies.^20^, ^21^, ^22^ Briefly, patients who received catheter ablation of AF had their antiarrhythmic medications discontinued for more than five half‐lives before the procedure, except for amiodarone. During the procedure, a 7‐French deflectable decapolar catheter with a 2‐mm interelectrode distance and 5‐mm space between each electrode pair (St. Jude Medical, St. Paul, MN, United States) was placed in the coronary sinus (CS) through an access in the right internal jugular vein. A high‐density mapping catheter (PentaRay, Biosense Webster, Diamond Bar, CA, United States; Orion, Boston Scientific, Cambridge, MA, United States; Spiral‐SC, St. Jude Medical) was coursed inside the LA via femoral access after the transseptal atrial puncture. Electroanatomic geometry and preablation and postablation voltage mapping of the LA was constructed using one of the three available three‐dimensional (3D) electroanatomic mapping systems: CARTO 3 (Biosense Webster), EnSite NavX (St. Jude Medical), or Rhythmia (Boston Scientific).

After subsequent geometry creation and mapping, circumferential pulmonary vein (PV) isolation was performed. Bilateral PVs were ablated circumferentially, with lesions encircling the antral side of both PV antra. An open irrigated‐tip force‐sensing ablation catheter (SmartTouch, Biosense Webster; TactiCath, St. Jude Medical; INTELLANAV STABLEPOINT, Boston Scientific) was used to ablate the PVs in a wide antral approach. PV isolation was successful after determining the entrance and exit blocks of all PVs and achieving the absence of any electrical activity inside the PVs or determining if there is the presence of dissociated electrical activity within the PVs during SR.

In cases of persistent AF, ablation strategies include linear and driver ablation. Successful ablation of persistent AF was achieved after ensuring entrance and exit blocks and the absence of any electrical activity along areas of identified focal or rotor activity after remapping the LA. If AF persisted despite extensive ablation, externally synchronized cardioversion using 100 or 200 Joules was deemed a last resort to convert AF back to SR.^23^

During SR or after the restoration of SR, the activation sequences of the high right atrium (HRA), His‐bundle, and CS were evaluated for the location of non‐PV triggers. Spontaneous onset of ectopic beats was located, and then infusion of isoproterenol (up to 4 mcg/min for 5 min) was given to see the initiation of AF. The time difference between the HRA and His‐bundle area activation during SR and ectopy can help reveal the site of ectopy or trigger, differentiating between the superior vena cava (SVC), upper crista terminalis, and PVs.^24^ If the measurement between the HRA and His‐bundle area during SR and ectopy was <0 ms, it was suggestive of SVC ectopy. For mapping of non‐PV triggers from the LA, if the earliest activation site was near the left PV ostium or posterolateral portion of the mitral annulus, differential pacing of the distal CS was performed to differentiate the ectopic beats from the vein of Marshall.^25^ The possibility of a vein of Marshall ectopy should be considered when triple potentials are recorded around the PV ostium. All non‐PV triggers and PV triggers were identified by positioning a mapping catheter in these areas. If an ectopic beat from this area consistently induced AF, it was then identified as a trigger.

### 2.5 Outcome assessment

All patients were followed up 1–2 weeks after AF ablation and every 3 months thereafter. The postablation follow‐up included resting surface 12‐leads electrocardiograms, transthoracic echocardiography, 24‐h Holter recordings, and/or cardiac event recording with a recording duration of 1 week performed regularly every 3 months for 1 year and an additional check‐up when patients reported clinical symptoms. One year after procedures, patients received regular follow‐up visits every 3–6 months.

### 2.6 Statistical analysis

Patient characteristics are expressed as mean ± standard deviation for continuous variables and as frequency (percentage) for categorical variables. Continuous and categorical variables were compared using the Student‐*t* test and the *χ* ^2^test with Yates' correction, respectively. Proportions were compared using the *χ* ^2^test or Fisher's exact test, as appropriate. One‐way analysis of variance was used to compare data among two groups. Univariable analysis was performed with a logistic regression model, and variables with *p* < .05 were taken into further multivariable analysis. Multivariable analysis was performed with a logistic regression model, with results expressed as odds ratios (ORs) with 95% confidence intervals (95% CIs). The final statistical significance was set at *p* < .05. Statistical analyses were performed using IBM SPSS 20 for Windows (SPSS, Inc., Chicago, IL).

## 3 RESULTS

### 3.1 Baseline characteristics

Our study included 84 patients with a mean age of 62.3 ± 9.4 years, 13.1% of whom were female. The average time from the initial echocardiography to the ablation procedure was 3.8 months, with the first quartile at 0.9 months and the third quartile at 4.3 months. All these patients with LVEF below 50% underwent AF ablation between 2019 and 2021. The median follow‐up time for echocardiography in our cohort was 7.8 months, with the first and third quartiles being 4.2 and 15.9 months, respectively. During the follow‐up, 68 (81%) patients had a recovery of LVEF, and we labeled them as responders (Figure 1). The body mass index (BMI) (25.7 ± 3.3 vs. 25.5 ± 4.4 kg/m^2^; *p* = .847), CHA_2_DS_2_‐VASc score (2.4 ± 1.3 vs. 2.5 ± 1.4 points; *p* = .376), AF pattern (paroxysmal AF 32.4% vs. 43.8%; *p* = .388), underlying comorbidities and the use of HF medications/ADDs and were similar between the two groups, as summarized in Table 1.

**TABLE 1:** Baseline characteristics of the study population.

	Total (*n* = 84)	Responder (*n* = 68)	Nonresponder (*n* = 16)	*p value*
Age (years)	62.3 ± 9.4	62.4 ± 9.1	61.8 ± 11.0	.802
Male gender, *n* (%)	73 (86.9%)	59 (86.8%)	14 (87.5%)	.937
BMI (kg/m^2^)	25.7 ± 3.5	25.7 ± 3.3	25.5 ± 4.4	.847
QRS (ms)	102.8 ± 21.8	99.7 ± 19.3	116.1 ± 27.0	**.006**
CHA2DS2‐VASc score	2.9 ± 1.4	2.9 ± 1.4	2.8 ± 1.1	.588
Follow‐up time (days)	243 ± 109	253 ± 107	202 ± 114	.085
Co‐morbidity, *n* (%)				
Hypertension	41 (48.8%)	34 (50.0%)	7 (43.8%)	.653
Diabete mellitus, type 2	16 (19.0%)	12 (17.6%)	4 (25.0%)	.500
Dyslipidemia	17 (20.5%)	15 (22.1%)	2 (13.3%)	.448
Coronary artery disease	25 (29.8%)	18 (26.5%)	7 (43.8%)	.174
Stroke	8 (9.6%)	8 (11.8%)	0 (0%)	.162
Thyroid disease	2 (2.4%)	2 (2.9%)	0 (0%)	.501
Baseline echocardiography				
LA volume index (mL/m^2^)	39.3 ± 14.6	37.3 ± 12.4	47.7 ± 20.2	**.010**
LVIDd (mm)	52.3 ± 7.7	51.5 ± 7.5	56.2 ± 7.9	**.036**
LVIDs (mm)	40.7 ± 8.2	39.9 ± 8.1	44.6 ± 7.7	.053
IVSd (mm)	10.4 ± 2.1	10.3 ± 1.8	10.8 ± 2.9	.529
LVPWd (mm)	10.5 ± 1.9	11.0 ± 2.7	10.4 ± 1.7	.449
LVESV (mL)	55.7 ± 25.9	65.0 ± 31.9	53.7 ± 24.2	.139
LVEDV (mL)	92.3 ± 37.3	108.3 ± 51.5	88.8 ± 33.0	.193
LVEF (%)	39.2 ± 8.1	38.8 ± 8.6	40.7 ± 5.6	.305
Severe LV systolic dysfunction (LVEF <40%)	35 (41.7%)	30 (44.1%)	5 (31.3%)	.348
PASP (mmHg)	31.8 ± 10.4	30.8 ± 8.7	36.1 ± 15.6	.241
Medication of heart failure, *n* (%)				
ACE‐I/ARB/ARNI	45 (53.6%)	34 (50.0%)	11 (68.8%)	.176
Beta‐blocker	52 (61.9%)	39 (57.4%)	13 (81.3%)	.077
SGLT‐2 inhibitor	22 (26.2%)	15 (22.1%)	7 (43.8%)	.076
Amiodarone	41 (48.8%)	34 (50.0%)	7 (43.8%)	.653
Digoxin	10 (11.9%)	8 (11.8%)	2 (12.5%)	.935
Parameter of the ANTWERP score, *n* (%)				
Severe atrial dilation (LAVI >50 mL/m^2^)	18 (21.4%)	11 (16.7%)	7 (43.8%)	**.016**
QRS >120 ms	12 (14.3%)	7 (10.3%)	5 (31.3%)	**.031**
Paroxysmal AF	55 (65.5%)	46 (67.6%)	9 (56.3%)	.388
Known etiology of cardiomyopathy	24 (28.6%)	14 (20.6%)	10 (62.5%)	**.001**
Etiology of known cardiomyopathy				
Ischemic cardiomyopathy	18 (21.4%)	13 (19.1%)	5 (31.3%)	.287
Dilated cardiomyopathy	3 (3.6%)	0 (0%)	3 (18.8%)	**<.001**
Hypertrophic cardiomyopathy	1 (1.2%)	1 (1.5%)	0 (0%)	.626
Valvualr heart disease	1 (1.2%)	0 (0%)	1 (6.3%)	**.038**
Fabry disease	1 (1.2%)	0 (0%)	1 (6.3%)	**.038**
ANTWERP score	1.4 ± 1.4	1.1 ± 1.2	2.8 ± 1.7	**<.001**
ANTWERP score 0–2, *n* (%)	66 (78.6%)	58 (85.3%)	8 (50%)	**.002**
ANTWERP score 3–4, *n* (%)	15 (17.9%)	10 (14.7%)	5 (31.3%)	.120
ANTWERP score ≥5, *n* (%)	3 (3.6%)	0 (0%)	3 (18.8%)	**<.001**
Ablation characteristics				
Procedure time (min)	231.6 ± 65.4	224 ± 57.7	233.4 ± 67.4	.612
Radiation time (min)	46.2 ± 33.0	51.8 ± 33.8	44.6 ± 32.9	.476
PVI, *n* (%)	84 (100.0%)	68 (100.0%)	16 (100.0%)	
Non‐PV ablation, *n* (%)				
Trigger ablation	32 (38.1%)	24 (35.3%)	8 (50.0%)	.276
Linear ablation	18 (21.4%)	13 (19.1%)	5 (31.3%)	.287
CTI ablation	65 (77.4%)	55 (80.9%)	10 (62.5%)	.114
Complication, *n* (%)				
Vascular complication	0 (0.0%)	0 (0.0%)	0 (0.0%)	
Pericardial effusion	0 (0.0%)	0 (0.0%)	0 (0.0%)	
Postablation AAD, *n* (%)				
Class I	19 (22.6%)	15 (22.1%)	4 (25.0%)	.800
Class II	37 (44.0%)	31 (45.6%)	6 (37.5%)	.558
Class III	31 (36.9%)	23 (33.8%)	8 (50.0%)	.228
Class IV	0 (0.0%)	0 (0.0%)	0 (0.0%)	

*Note*: The statistical significance was set at *p* < 0.05 and was in bold values.

Abbreviations: AAD, Anti‐arrhythmic drug; BMI, body mass index; CTI, cavotricuspid isthmus; IVSd, interventricular septum thickness at end‐diastole; LA, left atrium; LV, left ventricular; LVEDV, LV end‐diastolic volume; LVEF, left ventricular ejection fraction; LVESV, LV end‐systolic volume; LVIDd, LV internal diameter at end‐diastole; LVIDs, LV internal diameter at end‐systole; LVPWd, LV posterior wall thickness at end‐diastole; PASP, pulmonary artery systolic pressure; PVI, Pulmonary vein isolation; SGLT‐2, sodium‐glucose cotransporter‐2.

Baseline echocardiographic parameters between responders and nonresponders are also detailed in Table 1. The baseline LVEF exhibited no significant variation between groups (38.8 ± 8.6 vs. 40.7% ± 5.6%; *p* = .305). Pulmonary artery systolic pressure (PASP) (30.8 ± 8.7 vs. 36.1 ± 15.6 mmHg; *p* = .241) was higher in nonresponders, albeit without significant difference. A statistically significant difference was observed in the left atrial volume index (LAVI) (37.4 ± 12.3 vs. 47.2 ± 20.8 mL/m^2^; *p* = .010) and the left ventricular internal diameter in diastole (LVIDd) (51.5 ± 7.5 vs. 56.2 ± 7.9 mm; *p* = .036).

Of the 24 patients with known etiologies of cardiomyopathy, there was no difference between patients with ischemic cardiomyopathy (19.1% vs. 31.3%, *p* = .287), and patients with hypertrophic cardiomyopathy (1.5% vs. 0%, *p* = .120).

There are statistical significance between the two groups in patients with dilated cardiomyopathy (0% vs. 18.8%, *p* < .001), valvular heart disease previously undergone mitral valve replacement (0% vs. 6.3%, *p* = .038), and patient with Fabry disease (0% vs. 6.3%, *p* = .038).

### 3.2 Structural remodeling after ablation of AF

**FIGURE 2:**
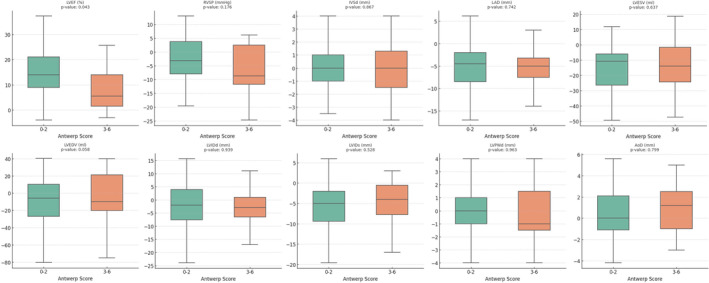
Comparison of baseline and follow‐up echocardiography parameters in (A) patients with ANTWERP Score 0–2 and (B) patients with ANTWERP Score 3–6. LV, Left ventricular; LAD, Left atrium diameter; LVIDd, LV internal diameter at end‐diastole; LVIDs, LV internal diameter at end‐systole; IVSd, Interventricular septum thickness at end‐diastole; LVPWd, LV posterior wall thickness at end‐diastole; LVESV, LV end‐systolic volume; LVEDV, LV end‐diastolic volume; LVEF, Left ventricular ejection fraction; PASP, Pulmonary artery systolic pressure.

More than 80% (*n* = 68) of the patients were classified as Responders during an average 8 months of follow‐up with echocardiography. The cardiac remodeling after ablation revealed by echocardiography is shown in Table 2. Responders had more significant ventricular reversed remodeling than nonresponders, as improved LVIDs and LVEF demonstrated, while left atrial remodeling was similar. For patients with ANTWERP score ≤2, 87.8% were responders, while only 55.6% of patients with ANTWERP score >2 were responders. A comparison of the pre‐ablation echocardiographic parameters according to different ANTWERP score categories (Score 0–2 vs. Score 3–6) is listed in Table 3, where only the LAVI (36.8 ± 13.0 vs. 48.2 ± 17.3 mL/m^2^; *p* = .019) and PASP (30.2 ± 9.0 vs. 37.6 ± 13.3 mmHg; *p* = .049) showed significant differences. The paradigm shift between the two groups (Score 0–2 vs. Score 3–6) is illustrated in Figure 2. A significant difference was noted in the change in LVEF (Delta LVEF) (14.8 ± 9.6 vs. 9.4% ± 11.0%; *p* = .043). The ventricular reversed remodeling revealed by LVEDV was also noted in patients with Score 0–2, though the change did not reach statistical significance.

**TABLE 2:** Comparative analysis of echocardiographic parameter changes between responders and nonresponders.

	Responder (*n* = 68)	Nonresponder (*n* = 16)	*p‐value*
LAD (mm)	−5.2 ± 5.7	−3.8 ± 4.8	.485
LVIDd (mm)	−2.4 ± 7.4	−1.4 ± 9.8	.719
LVIDs (mm)	−6.8 ± 6.9	−1.5 ± 10.5	.061
IVSd (mm)	0.2 ± 1.9	0.3 ± 2.1	.948
LVPWd (mm)	−0.1 ± 1.6	−0.2 ± 3.4	.910
LVESV (mL)	−17.0 ± 26.6	−5.2 ± 27.1	.241
LVEDV (mL)	0.6 ± 88.4	−5.7 ± 51.6	.846
LVEF (%)	16.6 ± 8.6	0.4 ± 4.6	**<.001**
PASP (mmHg)	−3.4 ± 9.8	−6.3 ± 10.0	.455

*Note*: The statistical significance was set at *p* < 0.05 and was in bold values.

Abbreviations: IVSd, interventricular septum thickness at end‐diastole; LAD, left atrium diameter; LV, left ventricular; LVEDV, LV end‐diastolic volume; LVEF, left ventricular ejection fraction; LVESV, LV end‐systolic volume; LVIDd, LV internal diameter at end‐diastole; LVIDs, LV internal diameter at end‐systole; LVPWd, LV posterior wall thickness at end‐diastole; PASP, pulmonary artery systolic pressure.

**TABLE 3:** Comparative analysis of pre‐ablation echocardiographic parameters across various ANTWERP score categories.

	Antwerp score 0–2 (*n* = 66)	Antwerp score 3–6 (*n* = 18)	*p‐value*
LA volume index (mL)	36.8 ± 13.0	48.2 ± 17.3	**.019**
LVIDd (mm)	51.7 ± 7.7	54.8 ± 7.4	.150
LVIDs (mm)	40.4 ± 7.9	52.1 ± 9.1	.448
IVSd (mm)	10.2 ± 2.0	11.3 ± 2.2	.051
LVPWd (mm)	10.4 ± 2.0	10.8 ± 1.9	.416
LVESV (mL)	53.5 ± 25.6	64.3 ± 26.1	.138
LVEDV (mL)	89.3 ± 35.5	103.9 ± 43.0	.116
LVEF (%)	39.6 ± 8.6	37.7 ± 8.5	.396
PASP (mmHg)	30.2 ± 9.0	37.6 ± 13.3	**.049**

*Note*: The statistical significance was set at *p* < 0.05 and was in bold values.

Abbreviations: IVSd, interventricular septum thickness at end‐diastole; LA, left atrium; LV, left ventricular; LVEDV, LV end‐diastolic volume; LVEF, left ventricular ejection fraction; LVESV, LV end‐systolic volume; LVIDd, LV internal diameter at end‐diastole; LVIDs, LV internal diameter at end‐systole; LVPWd, LV posterior wall thickness at end‐diastole; PASP, pulmonary artery systolic pressure.

Overall, patients with lower left ventricular ejection fraction (LVEF <40%) demonstrated a significantly greater improvement in LVEF compared to those with only mildly reduced LVEF (LVEF ≥40% and <50%) (20.0% vs. 9.2%, *p* < .001). We further analyzed LVEF recovery between two groups based on the ANTWERP score: patients with scores ≤2 points and those with scores >2 points. Within these groups, patients with lower LVEF continued to show significantly greater improvement compared to those with mildly reduced LVEF. In the ANTWERP score ≤2 points group, patients with lower LVEF exhibited a 22.6% improvement versus 10.3% (*p* < .001). In the ANTWERP score >2 points group, patients with lower LVEF showed a 13.9% improvement compared to 3.7% in the mildly reduced LVEF group (*p* = .037).

### 3.3 Predictors of LV function improvement

Univariate analysis of our study group (refer to Table 4) showed significant associations between left ventricular function recovery and the following: LAVI with an OR of 0.95 (95% CI: 0.92–0.99, *p* = .015), QRS duration with an OR of 0.97 (95% CI: 0.95–0.99, *p* = .012), and the ANTWERP score with an OR of 0.46 (95% CI: 0.30–0.71, *p* = <.001). Multivariate analysis (also in Table 4) further determined that the ANTWERP score was independently associated with an improvement in LVEF, according to the “Universal Definition and Classification of HF,” with an OR of 0.52 (95% CI: 0.30–0.90, *p* = .020). We performed the ROC analysis of the AF prediction with ANTWERP score, and the AUC was 0.8 [95% CI 0.686–0.905].

**TABLE 4:** Predictors of LV function improvement (*n* = 84).

	Univariant analysis		Multivariant analysis	
	OR (95% CI)	*p*	OR (95% CI)	*p*
Age (years)	1.01 (0.95–1.07)	.800		
Male gender	0.94 (0.18–4.82)	.937		
BMI (kg/m^2^)	1.02 (0.87–1.19)	.845		
CHA2DS2‐VASc score	1.23 (0.78–1.92)	.372		
Hypertension	1.29 (0.43–3.85)	.653		
Diabete mellitus, type 2	0.64 (0.18–2.34)	.503		
Coronary artery disease	0.68 (0.39–1.19)	.180		
Congestive heart failure	1.73 (0.47–6.36)	.411		
LAVI (mL/m^2^)	0.95 (0.92–0.99)	**.015**	0.98 (0.94–1.02)	.331
IVSd (mm)	0.88 (0.67–1.15)	.351		
LVEDV (mL)	0.99 (0.97–1.00)	.087		
LVEF (%)	0.97 (0.90–1.04)	.420		
PASP (mmHg)	0.96 (0.91–1.01)	.124		
QRSd (ms)	0.97 (0.95–0.99)	**.012**	1.00 (0.97–1.03)	.783
Paroxysmal AF	0.62 (0.20–1.87)	.391		
ANTWERP score	0.46 (0.30–0.71)	**<.001**	0.52 (0.30–0.90)	**.020**

*Note*: The statistical significance was set at *p* < 0.05 and was in bold values.

Abbreviations: AF, atrial fibrillation; LAVI, left atrium volume index; LV, left ventricular; LVIDd, LV internal diameter at end‐diastole; QRSd, QRS duration.

### 3.4 Comparison of AF recurrence within 1 year

**FIGURE 3:**
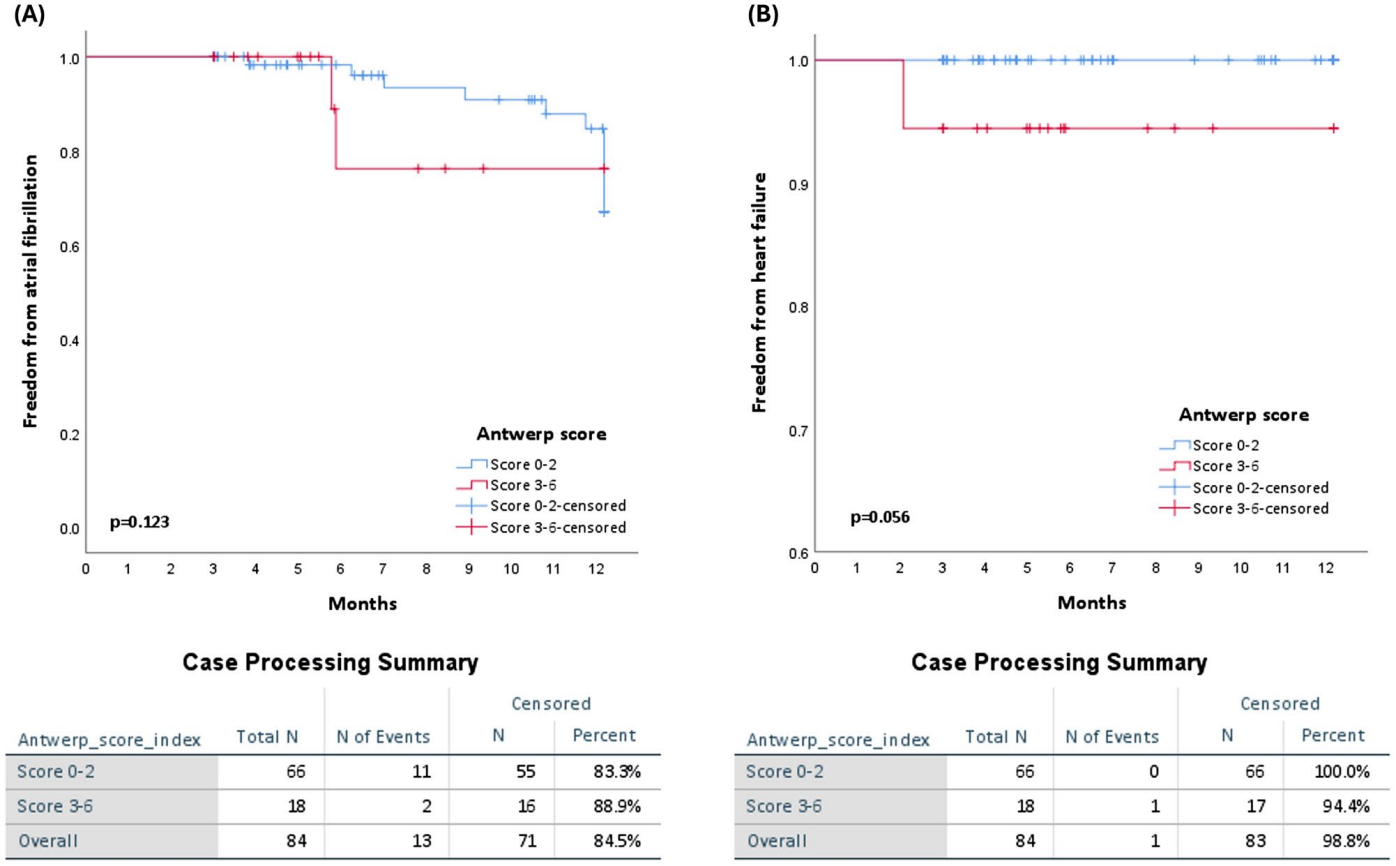
Comparison of atrial arrhythmia recurrence between patients with ANTWERP Score 0–2 and ANTWERP Score 3–6. AF, Atrial fibrillation. (A) Kaplan–Meier analysis depicting the cumulative event rates for the composite endpoints of atrial arrhythmia recurrence in two groups: ANTWERP Score 0–2 and ANTWERP Score 3–6. (B) Kaplan–Meier analysis depicting the cumulative event rates for the composite endpoint of the heart failure hospitalization in two groups: ANTWERP Score 0–2 and ANTWERP Score 3–6.

Overall, no significant procedural complications were observed. During regular follow‐up, 13 patients (15.4%) reported recurrences of atrial arrhythmia. These findings are depicted in Figure 3A. Patients with Score 0–2 had 16.7% recurrence of atrial arrhythmia (9.1% recurrence in PAF patients and 90.9% recurrence in PerAF patients), while patients with Score 3–6 had 11.1% recurrence of atrial arrhythmia (0% recurrence in PAF patients and 100% recurrence in PerAF patients). There was no difference in recurrent atrial arrhythmia between patients with scores of 0–2 and 3–6, as summarized in Table 5 (*p* = .563).

**TABLE 5:** Characteristics between patients with or without atrial fibrillation recurrence.

	No AF recurrence (*n* = 71)	AF recurrence (*n* = 13)	*p‐value*
Age (years)	62.8 ± 9.2	59.6 ± 10.6	.329
Male gender, *n* (%)	61 (85.9%)	12 (92.3%)	.530
BMI (kg/m^2^)	25.7 ± 3.3	25.6 ± 4.6	.902
CHA2DS2‐VASc score	3.0 ± 1.4	2.3 ± 1.3	.107
Co‐morbidity, *n* (%)			
Hypertension	36 (50.7%)	5 (38.5%)	.417
Diabetes mellitus, type 2	14 (19.7%)	2 (15.4%)	.714
Dyslipidemia	16 (22.5%)	1 (7.7%)	.221
Coronary artery disease	23 (32.4%)	2 (15.4%)	.217
Stroke	8 (11.3%)	0 (0%)	.203
Parameter of the ANTWERP score, *n* (%)			
Severe LV systolic dysfunction (LVEF <40%)	30 (42.3%)	5 (38.5%)	.799
Severe atrial dilation (LAVI > 50 mL/m^2^)	16 (22.5%)	2 (15.4%)	.563
QRS > 120 ms	11 (15.5%)	1 (7.7%)	.460
Known etiology of cardiomyopathy	21 (29.6%)	3 (23.1%)	.633
Paroxysmal AF	46 (67.6%)	9 (56.3%)	.388
ANTWERP Score	1.52 ± 1.4	0.9 ± 1.6	.121
ANTWERP score 0–2	55 (77.5%)	11 (84.6%)	.563
ANTWERP score 3–4	14 (19.7%)	1 (7.7%)	.298
ANTWERP score ≥5	2 (2.8%)	1 (7.7%)	.384

Abbreviations: BMI, body mass index; LAVI, left atrium volume index; LV, left ventricular; LVEF, left ventricular ejection fraction.

### 3.5 Comparison of major adverse cardiac events

During our cohort's follow‐up period, only one patient experienced hospitalization because of HF. We have provided the Kaplan–Meier curve for HF hospitalization, and there was no significant difference between patients with scores of 0–2 and 3–6 (Figure 3B). The patient who experienced HF exacerbation 62 days post‐AF ablation had an ANTWERP score of 5 and a CHA₂DS₂‐VASc score of 4. Furthermore, there were no reported cases of mortality and stroke throughout the follow‐up period.

## 4 DISCUSSION

### 4.1 Main findings

This study shows that the ANTWERP score can reliably forecast the LVEF recovery in an Asian group. Patients with ANTWERP score ≤2 had more improved ventricular remodeling. The rate of recurrent atrial arrhythmia was comparable between responders and nonresponders.

### 4.2 Previous studies of AF ablation in patients with systolic dysfunction

Previous systematic reviews and meta‐analyses demonstrated a decrease in all‐cause mortality and HF hospitalizations with AF ablation in comparison to medical therapy.^7^, ^8^, ^26^, ^27^, ^28^ The favorable outcomes reported with catheter ablation could be attributed to improved LVEF and reduced AF burden. However, only a variable percentage (9%–68%) of patients with HF experience LVEF improvement after AF ablation. The AMICA trial also demonstrated comparable enhancements in LVEF, 6‐min walk test performance, and quality of life between groups receiving catheter ablation and those undergoing medical therapy. No guidance could be applied to identify those who will most likely have improved LVEF.^29^, ^30^

Although several studies have been made to identify predictors of LVEF recovery after ablation,^9^, ^24^, ^25^, ^26^ the small sample size and the heterogeneous study cohorts are limitations. Nevertheless, etiology, AF pattern, LVEF, dilated LV, and older age have been reported as predictors of LVEF recovery after AF ablation.^29^, ^31^, ^32^, ^33^, ^34^ In our results, patients with ANTEWERP score ≤2 have better LVEF improvement and reversed remodeling than those with ANTEWERP score >2, while the atrial reverse remodeling and recurrent atrial arrhythmia were similar between the two groups.

### 4.3 Ethnicity and HF and AF outcomes

Previous studies have shown ethnic differences in outcomes for adults with HF. Compared to White patients, Black patients had more HF hospitalizations but less mortality, while Asian patients had similar HF hospitalizations but less all‐cause hospitalizations and mortality.^35^, ^36^ AF increases the risk of many adverse events, such as stroke, HF, and death. Ethnic differences in the impact of AF on these events have also been reported. For example, among older patients with AF enrolled in Medicare, non‐Hispanic Black patients had higher mortality and stroke rates than non‐Hispanic White patients.^37^ Similarly, Black and Hispanic patients with AF had higher stroke rates than White patients in another database analysis.^38^ For AF ablation in HF patients, the CABANA trial showed that ablation was better than drug therapy for racial or ethnic minorities in the North American cohort.^39^ In our results, AF ablation also improved heart function in some Asian HF patients. Patients with an ANTWERP score ≤2 had a high chance (87.9%) of LVEF improvement after AF ablation, but patients with an ANTWERP score >2 had a low chance (55.6%). Our results showed that AF recurrence was similar between responders and nonresponders (Figure 3A). In the CASTLE‐AF trial, AF recurrence did not affect mortality and HF hospitalization after ablation.^4^ However, an AF burden <50% after a procedure predicted better outcomes in HF patients with AF, including lower mortality and HF hospitalization rates. These findings suggest that reducing AF burden is more important than preventing AF recurrence in managing AF in HF patients. Our results indicated that the new ANTWERP score could predict LVEF improvement after AF ablation in Asian HF patients, regardless of AF recurrence.

### 4.4 Gender distribution discrepancy

In our cohort, males predominate, comprising 86.9% of the participants, with females representing only 13.1%. This gender distribution does not reflect the typical gender parity seen in patients with AF.^40^ Studies have shown that women with AF tend to be older and have more comorbidities yet are less likely to receive rhythm control treatments such as electrical cardioversion or ablation compared to their male counterparts.^41^ Additionally, HFrEF is less common in women than in men.^42^ These factors may contribute to the observed gender disparity in our study.

### 4.5 Study limitations

First, the case number in this study was relatively small. Additional prospective, randomized studies involving a larger cohort are necessary to investigate the accuracy of ANTWERP score in AF ablation in patients with HF. Second, evaluating asymptomatic episodes of AF was difficult, even when Holter or cardiac event recorders were used. Third, some patients were followed with a 24‐hours Holter monitor, while others were followed with 1‐week recordings of a cardiac event recorder. The difference in ECG monitoring during follow‐up and the duration of follow‐up between the two groups might impact the recurrence. Finally, due to the retrospective nature of our data collection and our status as a tertiary referral center, our capacity to obtain adequate information on echocardiography previously performed at other centers is limited. Additionally, most of the missing echocardiography data pertain to preoperative assessments.

## 5 CONCLUSION

The study found that the ANTWERP score accurately predicted LVEF improvement after AF ablation in the Asian population and that this scoring system could help with clinical decision‐making and prognosis prediction. The ANTWERP score has shown a strong predictive ability for LVEF improvement in Asian patients who underwent AF ablation, especially those with LVEF <50%. The results suggest that patients with an ANTWERP score ≤2 are more likely to have significant LVEF improvement and more favorable ventricular remodeling than those with a score >2. Although the rates of atrial arrhythmia recurrence were similar across different score groups, the ANTWERP score's usefulness in guiding clinical choices and expectations for HF improvement after AF ablation is clear.

## AUTHOR CONTRIBUTIONS

Conceptualization, T.‐Y.C. and Y.‐J.L.; methodology, S.‐L.C.; software, C.‐Y.L.; validation, S.‐L.C., Y.‐J.L., and L.‐W.L.; formal analysis, L.‐C.L.; investigation, F.‐P.C. and Y.‐F.H.; resources, Y.‐F.H.; data curation, T.‐Y.C.; writing—original draft preparation, L.‐C.L.; writing—review and editing, T.‐Y.C.; visualization, C.‐Y.L. and F.‐P.C.; supervision, L.‐W.L.; project administration, S.‐A.C.; funding acquisition, Y.‐J.L. and S.‐A.C. All authors have read and agreed to the published version of the manuscript.

## FUNDING INFORMATION

This study was supported, in part, by research grants from the Taipei Veterans General Hospital (V108C‐055, V109C‐008, V110C‐004, and V111C‐075), Ministry of Science and Technology of Taiwan (MOST106‐2314‐B‐010‐035‐MY3, 109‐2314‐B‐010‐063, 110‐2314‐B‐A49A‐542, and 111‐2314‐B‐075‐007‐MY3), SZU‐YUAN RESEARCH FOUNDATION OF INTERNAL MEDICINE (108010, 109024, 110008, and 111003), and “Yin Yen‐Liang Foundation Development and Construction Plan” of the College of Medicine, National Yang Ming Chiao Tung University.

## CONFLICT OF INTEREST STATEMENT

Authors declare no conflict of interests for this article.

## ETHICS STATEMENT

This retrospective study was conducted at the Taipei Veterans General Hospital in Taipei after approval by the institutional review board of the Taipei Veterans General Hospital (IRB: 2022‐06‐001BC).

## INFORMED CONSENT

Informed consent was obtained from all subjects involved in the study.

## Data Availability

The data presented in this study are not publicly available due to upcoming publications but are available at the request of the corresponding author.
